# Lung Function Assessment Among Patients Previously Treated for Pulmonary Tuberculosis at Tikur Anbessa Specialized Hospital, Addis Ababa, Ethiopia

**DOI:** 10.4314/ejhs.v35i1.9S

**Published:** 2025-12

**Authors:** Amsalu Bekele

**Affiliations:** 1 Division of Pulmonary and Critical Care Medicine, Department of Internal Medicine, College of Health Sciences, Addis Ababa University

**Keywords:** Pulmonary tuberculosis, Lung function, Previous TB treatment

## Abstract

**Background:**

Lung function impairment is common among patients who have been successfully treated (cured or treatment completed) for pulmonary tuberculosis (PTB). However, post-treatment lung function outcomes in Ethiopia remain poorly characterized. The objective of this study was to evaluate pulmonary function in patients successfully treated for PTB at Tikur Anbessa Specialized Hospital (TASH) in Ethiopia.

**Methods:**

This cross-sectional study included patients older than 15 years who had been successfully treated for PTB and were followed at the Chest Unit of TASH between August 2016 and September 2017. Patients with active PTB were excluded. Sociodemographic characteristics, PTB diagnosis and treatment history, smoking status, and biomass fuel exposure were obtained from clinic records and patient interviews. All participants underwent spirometry. Lung function was classified as normal or abnormal according to standard spirometric criteria.

**Results:**

A total of 99 patients were included in the analysis. Fifty-five (55.6%) were male, and the mean age was 42.7 years. Mean spirometric values were: FEV_1_ 53.9% predicted, FVC 63.1% predicted, and FEV_1_/FVC 59.2%. Spirometric patterns were classified as normal in 14 (14.1%) patients, obstructive in 41 (41.4%), restrictive in 42 (42.4%), and mixed in 2 (2.0%). Factors significantly associated with abnormal lung function included recurrent PTB, age >40 years, smoking history, and biomass exposure from cooking for more than 10 years (p < 0.05).

**Conclusion:**

The prevalence of abnormal lung function was high among Ethiopian patients successfully treated for PTB. We recommend close spirometric monitoring to assess progression of pulmonary impairment in this population.

## Introduction

Tuberculosis (TB) remains one of the ten leading causes of death globally and is the leading cause of death from a single infectious agent, surpassing HIV/AIDS. In 2016, an estimated 1.3 million TB-related deaths occurred among HIV-negative individuals, with an additional 374,000 deaths among people living with HIV (1). During the same year, approximately 10.4 million people developed active TB; 90% were adults, 65% were male, and 10% were people living with HIV, the majority of whom resided in Africa (1).

Pulmonary tuberculosis (PTB) can result in significant lung parenchymal destruction through upregulation of proteases and dysregulation of protease inhibition (2). Histopathological sequelae following PTB treatment includes fibrosis, bronchiectasis, and bronchial stenosis, which may lead to persistent pulmonary function abnormalities (3,4). These impairments can contribute to unemployment, increased healthcare utilization, reduced quality of life, and potentially shortened life expectancy.

Lung function impairment is frequently observed among patients who have been successfully treated for PTB (5,6). Longitudinal studies indicate that 49–76% of such patients exhibit permanent airflow obstruction, restrictive defects, or mixed ventilatory patterns (7–10). Consequently, post-tuberculosis pulmonary impairment has emerged as a distinct clinical entity (11–23).

In Ethiopia, data on pulmonary function following successful PTB treatment are limited. The primary objective of this study was to assess pulmonary function among patients successfully treated for PTB at Tikur Anbessa Specialized Hospital (TASH), the largest public tertiary hospital in Ethiopia. A secondary objective was to identify factors associated with post-treatment pulmonary function impairment.

## Methods and Materials

**Study design and population**: We conducted a hospital-based cross-sectional study in the Chest Unit of Tikur Anbessa Specialized Hospital (TASH) from August 2016 to September 2017. TASH is a tertiary referral hospital in Addis Ababa, Ethiopia, providing care for approximately 370,000–400,000 patients annually. The hospital comprises 16 outpatient clinics; the Chest Unit alone manages more than 500 patient visits per month, including individuals with a history of PTB.

The study population included consecutive patients aged over 15 years who had previously been successfully treated (cured or treatment completed) for PTB. Active TB was excluded using the World Health Organization systematic screening criteria (24). Data was collected through review of medical records and patient interviews using a structured questionnaire. Variables included sociodemographic characteristics, PTB diagnosis and treatment history, smoking status, and exposure to biomass fuels used for domestic cooking.

**Pulmonary function measurement**: Pulmonary function testing was performed using a Diagnostic EasyOne Plus Model 2001 SN spirometer by a trained technician. Spirometry acceptability and reproducibility were assessed according to European Respiratory Society and American Thoracic Society standards (25).

Based on spirometric results, lung function was categorized as normal or abnormal (obstructive, restrictive, or mixed) using the National Lung Health Education Program (NLHEP) algorithm (26). Obstruction was defined as an FEV_1_/FVC ratio below the lower limit of normal (LLN) with FVC ≥ LLN. Restriction was defined as an FEV_1_/FVC ratio ≥ LLN with FVC < LLN. A mixed pattern was defined by FEV_1_, FVC, and FEV_1_/FVC all below the LLN. Severity was graded using GOLD criteria for obstruction (27,28) and ATS/ERS recommendations for restriction (25).

**The following Operational definitions are used:**

⚬ **Forced vital capacity (FVC)**: Total volume of air forcefully exhaled after maximal inhalation.
⚬ **Forced expiratory volume in one second (FEV_1_)**: Volume of air exhaled during the first second of the FVC maneuver.
⚬ **FEV_1_/FVC ratio**: Proportion of air exhaled in the first second relative to total exhaled volume.
⚬ **Lower limit of normal (LLN)**: Lower boundary of normal lung function adjusted for age, sex, height, and race.
⚬ **Normal lung function**: FEV_1_/FVC, FEV_1_, and FVC ≥ LLN.
⚬ **Obstructive lung disease**: FEV_1_/FVC < LLN.
⚬ **Restrictive lung disease**: Reduced total lung capacity (<5th percentile of predicted) with normal or elevated FEV_1_/FVC ratio (ATS/ERS) (25).

**Data analysis**: Data were analyzed using SPSS version 20.0. Continuous variables were summarized using means ± standard deviation (SD) or medians with interquartile ranges (IQR), as appropriate. Categorical variables were expressed as frequencies and percentages. Spirometry variables (FEV_1_, FVC, and FEV_1_/FVC) were normally distributed, as assessed by visual inspection and the Shapiro-Wilk test (p = 0.064). Binary logistic regression was used to identify predictors of abnormal lung function. Independent variables included age, sex, educational level, smoking status, number of TB episodes, HIV serostatus, and biomass exposure. Statistical significance was defined as p < 0.05.

**Ethical approval:** Written informed consent was obtained from all participants. Ethical approval was granted by the Institutional Review Board of the College of Health Sciences, Addis Ababa University.

## Results

A total of 99 participants were enrolled in the study; sociodemographic characteristics are summarized in Table 1. Fifty-five participants (55.5%) were male, and the mean age was 42.7 years. Forty-nine participants (49.5%) were older than 40 years, and 62 (62.6%) had attained at least a primary level of education.

**Table 1:** Socio-demographic characteristics of the study population (N=99)

Characteristics	Frequency (%)
Age groups (years)	
15-25	12(12.1)
26-35	18(18.2)
36-45	29 (29.3)
46-55	25 (25.3)
56-65	9 (9.1)
>65	6 (6.1)
Sex	
Male	55 (55.6)
Female	44 (44.4)
Level of education	
Illiterate	37 (37.4)
Literate	62 (62.6)
Occupation	
Unemployed	16 (16.2)
Farming	12 (12.1)
Factory	6 (6.1)
Daily labor	5 (5.1)
Office	14 (14.1)
Housewife	16 (16.2)
Other	30 (30.3)
Smoking history	
Never smoke	83 (83.8)
Ever smoke	16 (16.2)
Passive smoker	7 (7.1)
Smoking pack years median (IQR)5.5(3-17.5)	
TB episode	
1X	68 (68.7)
≥2X	31 (31.3)
HIV Status:	
Positive	7 (7.1)
Negative	28 (28.3)
Unknown	64 (64.5)
History of Asthma	
Yes	16 (16.2)
No	83 (83.8)
Biomass exposure ≥ 10 years	
Yes	46 (46.5)
No	53 (53.5)

Forty-six participants (46.5%) reported exposure to biomass fuel, of whom 31 (31.3%) had been exposed for more than 10 years. Sixteen participants (16.2%) reported a history of smoking, and seven (7.0%) reported passive exposure to tobacco smoke. Most patients (68.7%) presented after their first episode of PTB, while 31 (31.3%) had experienced two or more episodes. Seven participants (7.1%) were HIV positive, 28 (28.3%) were HIV negative, and 64 (64.6%) had an unknown HIV serostatus.

**Figure 1:**
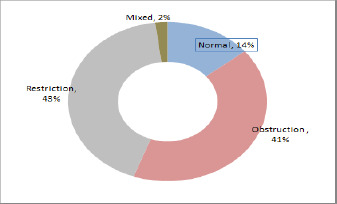
Lung function abnormality in study participants

The group's mean spirometry values were: FEV_1_ 53.9 % predicted, FVC 63.1% predicted, and FEV_1_/FVC 59.2%. Of the participants, 14(14.1%) had normal spirometry, 41 (41.1%) had obstructive spirometry, 42 (42.4%) had restrictive spirometry, and 2 (2.0%) % had a mixed pattern (Figure 1).

Significant factors associated with abnormal lung function were age ≥40 years (adjusted odds ratio [AOR] = 1.94; 95% confidence interval [CI]: 1.68–2.51; p = 0.049), a history of smoking (active or passive) (AOR = 1.47; 95% CI: 1.00–3.65; p = 0.049), biomass fuel exposure from cooking for ≥10 years (AOR = 3.53; 95% CI: 2.90–4.13; p = 0.03), and having more than one previous episode of PTB (AOR = 1.27; 95% CI: 1.04–1.64; p = 0.042) (Table 2).

**Table 2:** Socio-demographic factors associated with lung functions (N=99)

Characteristics (N =99)	Total	Normal PFT	Abnormal. PFT	COR (95% CI)	AOR (95% CI)	P-value
Age (year)						
<40	50	8	42	1	1	0.049
≥40	49	6	43	1.72(1.14-2.46)	1.94 (1.68-2.51)	
Active and passive smoking exposure						
Yes	21	2	19	2.18(1.15-2.98)	1.47 (1.00-3.65)	0.049
No	78	12	66	1	1	
Biomass fuel exposure (in year)						
≥10	23	2	21	3.12 (2.45-3.77)	3.53 (2.90-4.13)	0.03
<10	76	12	64	1	1	
Previous TB treatment history						
1x	68	7	62	1	1	0.042
2 or more	31	7	24	1.45(1.02-1.71)	1.27(1.04-1.64)	

Of the 99 patients, 85 (85.9%) had an FEV_1_, FVC, or FEV_1_/FVC ratio below the lower limit of normal (LLN). According to American Thoracic Society/European Respiratory Society criteria (25), the severity of impairment was classified as mild in 26 patients (26.2%), moderate in 19 (19.2%), severe in 21 (21.2%), and very severe in 19 (19.2%). Clinically significant pulmonary impairment—defined as an FEV_1_ <60% of the predicted value (15)—was identified in 59 patients (59.6%) (Table 3).

**Table 3:** Severity of obstructive lung function abnormality (FEV1) in terms of percent predicted in study participants

Lung function (FEV1%)	Number (%)
< 35% of predicted	19 (19.2)
35-49% of predicted	21 (21.2)
50-59% of predicted	19 (19.2)
60-69% of predicted	15 (15.2)
≥ 70% of predicted and < LLN	11 (11.0)
>70% of predicted and ≥ LLN	14 (14.0)

Further subgroup analyses were done to determine risk factors for obstructive and restrictive ventilatory disorders and to assess the additive effect of biomass exposure. Accordingly, these analyses showed no statistically significant associations (COR = 1.46, 95% CI: 0.75, 2.74, p-value = 0.267).

## Discussion

In our study, 85 (85.9%) patients successfully treated for PTB and followed in the Chest Unit of TASH demonstrated abnormal lung function, manifesting as restrictive (42.2%), obstructive (41.1%), or mixed (2.1%) ventilatory patterns. Moreover, most of these patients had moderate to severe impairment. These findings suggest that successfully treated PTB is a significant risk factor for reduced lung function and may contribute to the development of chronic lung disease, particularly in settings with a high tuberculosis burden where diagnosis and treatment may be delayed.

Although the magnitude of impairment observed in our study was somewhat higher, our findings are consistent with previously published research on post-PTB lung function. Mikhail and colleagues reported pulmonary function impairment in 102 (47.7%) patients in a similar study population (29). In that study, obstructive patterns were observed in 74 (34.6%) participants, and 60 (28.0%) had an FEV_1_ below the lower limit of normal. Manji and colleagues found abnormal lung function in 371 (72.7%) PTB patients in Dar es Salaam, with 42% demonstrating an obstructive pattern, 13% a restrictive pattern, and 19% a mixed pattern (30). In a case–control study by Pasipanodya and coworkers, 59% of tuberculosis patients had abnormal lung function compared with 20% of latent TB infection (LTBI) control subjects (22). Similarly, a study conducted in the United States found that 44% of PTB patients developed restrictive impairment, compared with 6.6% in the general population (23).

The higher prevalence of lung impairment observed in our study likely reflects a combination of more severe TB disease, delayed diagnosis, a higher burden of comorbidities (including HIV infection and malnutrition), and greater environmental exposures in Ethiopia compared with high-income countries (31,32). In addition, recruitment of patients from a specialized clinic at a tertiary referral center may partially explain the higher prevalence of abnormal lung function observed.

In our cohort, 69% of participants experienced a single episode of PTB, whereas 31% had two or more episodes. Abnormal lung function was more frequently observed among patients with recurrent PTB than among those with a single episode. This finding is consistent with the results of Hnizdo et al., who demonstrated a progressive decline in lung function with an increasing number of TB treatment courses (7).

Approximately 60% of participants in our study had moderate to severe lung function impairment. Such impairment is likely to contribute to reduced quality of life and increased healthcare utilization, particularly in low-resource settings. Routine spirometry screening of patients following successful PTB treatment may facilitate earlier identification of pulmonary impairment and allow timely management of chronic lung disease, thereby reducing long-term morbidity.

Abnormal lung function was significantly associated not only with recurrent PTB but also with age greater than 40 years, smoking history, and biomass fuel exposure exceeding 10 years. These findings are generally consistent with previous studies. Tobacco smoking and biomass smoke exposure are well-recognized risk factors for pulmonary function impairment, particularly airflow obstruction (33,34). Chung et al. reported that the history of PTB was a stronger determinant of impaired pulmonary function than cigarette smoking (35), suggesting that post-tuberculosis pulmonary inflammation may exacerbate smoking-related lung function decline. Mikhail and colleagues identified previous culture-positive TB, recurrent PTB, low educational level, and age greater than 50 years as predictors of reduced lung function (29). Similarly, Manji and coworkers found that abnormal lung function was associated with recurrent PTB, HIV-negative status, age over 40 years, and male sex (30).

Our inability to demonstrate associations between abnormal lung function and HIV status, male sex, or low educational level may be attributable to incomplete data and the relatively small sample size (3). The impact of HIV co-infection on lung function following PTB remains an important area for further investigation, particularly in regions such as Ethiopia where TB and HIV are highly prevalent. In our study, most participants had an unknown HIV serostatus, limiting definitive conclusions. Future studies incorporating confirmed HIV status and comprehensive pulmonary function testing are needed to clarify these relationships.

Spirometry is a widely used pulmonary function test for assessing lung function; however, it has important limitations in distinguishing true restrictive lung disease (RLD) from pseudo-restriction caused by air trapping in obstructive lung disease (OLD). This limitation arises because spirometry cannot measure total lung capacity (TLC), which is essential for confirming restriction. Several studies have demonstrated that spirometry restriction without lung volume measurement is frequently inaccurate. Schultz et al. (2023) evaluated 300 patients with spirometry restriction and found that only 36% had true RLD (TLC < 5th percentile); 40% had obstructive disease and 24% had nonspecific patterns with normal TLC and no obstruction (36). Analyses of NHANES data similarly indicate that epidemiologic studies relying solely on spirometry tend to overestimate restrictive disease by using FVC < LLN without TLC confirmation (37).

Because reductions in FVC may occur in obstructive disease, obesity, or neuromuscular disorders, spirometry alone is insufficient for diagnosing restrictive lung disease. Measurement of lung volumes, particularly TLC by plethysmography, is essential to differentiate true restriction from pseudo-restriction due to air trapping. Clinicians should therefore integrate spirometry with lung volume assessments for accurate interpretation of pulmonary function tests (36–38). Body plethysmography remains the gold standard for measuring TLC, residual volume (RV), and functional residual capacity (FRC), enabling precise classification of ventilatory defects. Studies comparing spirometry with plethysmography suggest that up to 40% of patients classified as restrictive by spirometry alone have normal TLC, highlighting the potential for misclassification (36–39).

Pulmonary function testing is not currently included in international guidelines for tuberculosis management (40). Our findings support incorporating lung function assessment into the follow-up of patients successfully treated for PTB to mitigate the development of chronic lung disease and potentially reduce the risk of TB recurrence.

This study has several limitations. The relatively small sample size limited statistical power to detect associations. Conducting the study at a tertiary referral center may have introduced selection bias. Only spirometry was available, precluding definitive diagnosis of restrictive lung disease due to the absence of lung volume measurements. Finally, single-center design limits generalizability; multicenter studies would provide more robust evidence.

In conclusion, abnormal lung function was highly prevalent among Ethiopian patients successfully treated for PTB. We recommend close spirometric monitoring to assess the progression of pulmonary impairment, particularly among individuals with identified risk factors. These findings contribute to the growing body of evidence that tuberculosis substantially increases the global burden of chronic lung disease.
